# Amyotrophic Lateral Sclerosis: Insights and New Prospects in Disease Pathophysiology, Biomarkers and Therapies

**DOI:** 10.3390/ph17101391

**Published:** 2024-10-18

**Authors:** Jameel M. Al-Khayri, Mamtha Ravindran, Akshatha Banadka, Chendanda Devaiah Vandana, Kushalva Priya, Praveen Nagella, Kowshik Kukkemane

**Affiliations:** 1 Department of Agricultural Biotechnology, College of Agriculture and Food Sciences, King Faisal University, Al-Ahsa 31982, Saudi Arabia; 2 Department of Biotechnology and Genetics, School of Sciences, JAIN (Deemed-to-be-University), Bangalore 560027, India; mamtha.r@jainuniversity.ac.in (M.R.); b.akshatha@jainuniversity.ac.in (A.B.); vandana.cd@jainuniversity.ac.in (C.D.V.); kushalvapriya@gmail.com (K.P.); 3 Department of Life Sciences, School of Sciences, Christ University, Bengaluru 560029, India; kowshik.k@christuniversity.in

**Keywords:** amyotrophic lateral sclerosis, pathology, neuroinflammation, diagnosis, therapies

## Abstract

Amyotrophic Lateral Sclerosis (ALS) is a severe neurodegenerative disorder marked by the gradual loss of motor neurons, leading to significant disability and eventual death. Despite ongoing research, there are still limited treatment options, underscoring the need for a deeper understanding of the disease’s complex mechanisms and the identification of new therapeutic targets. This review provides a thorough examination of ALS, covering its epidemiology, pathology, and clinical features. It investigates the key molecular mechanisms, such as protein aggregation, neuroinflammation, oxidative stress, and excitotoxicity that contribute to motor neuron degeneration. The role of biomarkers is highlighted for their importance in early diagnosis and disease monitoring. Additionally, the review explores emerging therapeutic approaches, including inhibitors of protein aggregation, neuroinflammation modulators, antioxidant therapies, gene therapy, and stem cell-based treatments. The advantages and challenges of these strategies are discussed, with an emphasis on the potential for precision medicine to tailor treatments to individual patient needs. Overall, this review aims to provide a comprehensive overview of the current state of ALS research and suggest future directions for developing effective therapies.

## 1. Introduction

Amyotrophic lateral sclerosis (ALS), also known as Lou Gehrig’s disease, is a progressive fatal neurodegenerative disease characterized by the loss of upper and lower motor neurons that weakens the muscles, ultimately resulting in paralysis [1]. Around 25% of patients experience bulbar symptoms such as slurred speech, difficulty swallowing, and weakness in facial muscles [2]. The prevalence is approximately 5/100,000 people/year globally, and on average the disease lasts for three years before demise [3]. However, 10% of patients may survive more than a decade [4]. ALS is most diagnosed in people between the ages of 58 and 60 [5]. Males are more susceptible to ALS than females, resulting in a male–female ratio of 1.2:1.5 [6]. A higher annual incidence rate is reported in Europe, at approximately 1–2 individuals/100,000 [7]. In contrast, results of population studies in East Asia and South Asia show incidence rates of0.8/100,000 individuals [8].

ALS is categorized broadly as familial and sporadic. Sporadic ALS (non-inherited) refers to cases where the disease appears in individuals without a known family history, whereas familial ALS (inherited) occurs when at least one of the family members is affected by the condition. Only about 10% of cases are familial (FALS), and the rest of them are sporadic [9]. Numerous genes have been linked to both sporadic and familial (inherited) forms of ALS. The most studied ALS-related genes include *C9orf72*, *SOD1*, *TARDBP* (*TDP-43*), and *FUS*, among others. Studying the roles of these genes in ALS pathogenesis is very important for developing targeted therapies and for better management of the disease.

**Figure 1:**
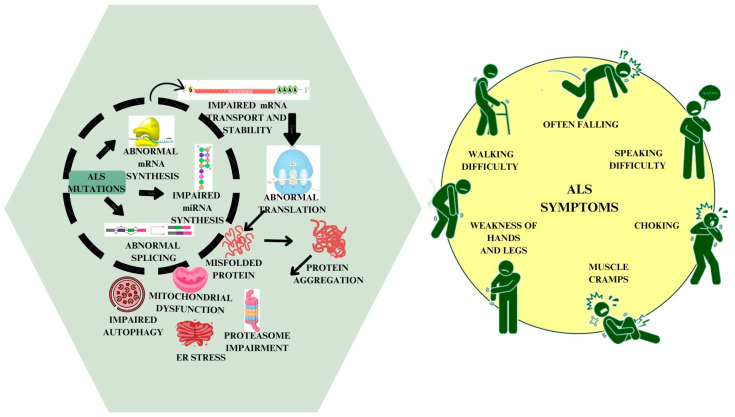
Comprehensive Overview of ALS: Diagnosis, Treatment, Pathophysiology, and Genetic Mutations.

ALS, being a fatal disease, has no cure, and current treatments only modestly slow its progression without stopping the eventual decline; therefore, developing effective therapeutics is critically important. The lack of a defined cause of the disease is mirrored in the number of therapeutics currently available. There are only two approved treatments for ALS despite previous and ongoing trials: Riluzole [10] and Edaravone, whose mechanism of action is unclear but assumed to be through their antioxidant properties [11]. These drugs marginally slow down the progression of the disease but do not cure it. Many novel therapeutics are entering clinical trials with the aim of finding more effective treatments and, ultimately, a cure. Figure 1 depicts the genetic mutations, pathophysiological mechanisms, diagnostic methods, and treatment strategies for ALS.

Although significant research has been conducted, the underlying cause of ALS remains unknown, and there is still no cure. This highlights the critical need for a deeper understanding of the disease and its progression. In light of this, the review aims to provide key insights into ALS pathology, the biomarkers used for diagnosis, and the therapies currently in use. By presenting this information, it seeks to offer a stronger foundation for developing novel and more effective treatments. As research continues to evolve, gaining a better understanding of ALS at both molecular and clinical levels could lead to promising therapeutic advancements.

## 2. Pathophysiology of ALS

ALS is a devastating neurodegenerative disorder characterized by the continuous degeneration of motor neurons in the brain and spinal cord [12]. In ALS, the endoplasmic reticulum (ER), mitophagy, and autophagy are main contributors to cellular dysfunction that leads to motor neuron degeneration. ER stress is a crucial component of ALS pathology and is responsible for protein folding, lipid synthesis, and calcium homeostasis. Protein misfolding and accumulation due to genetic mutations such as *SOD1* and *TDP-43* overload the ER, triggering ER stress. Persistent ER stress results in apoptosis and contributes to motor neuron death [13].

Mitophagy, the selective degradation of damaged mitochondria through autophagy, is essential for mitochondrial quality control. In ALS, dysfunctional mitophagy contributes to mitochondrial damage and impaired energy production, particularly in motor neurons. Mutations in genes like *C9orf72* and *OPTN* disrupt the mitophagic process, leading to the accumulation of defective mitochondria. This results in increased oxidative stress, calcium dysregulation, and neuronal energy deficits, all of which aggravate motor neuron damage [14].

Autophagy, the broader process of degrading and recycling damaged cellular components, is important for maintaining cellular homeostasis. In ALS, autophagic pathways are dysregulated, resulting in the accumulation of toxic aggregates, such as misfolded proteins and damaged organelles. This accumulation exceeds the cells’ capacity for degradation. This impaired autophagy contributes to cellular stress, protein aggregation, and ultimately the degeneration of motor neurons [15].

**Figure 2:**
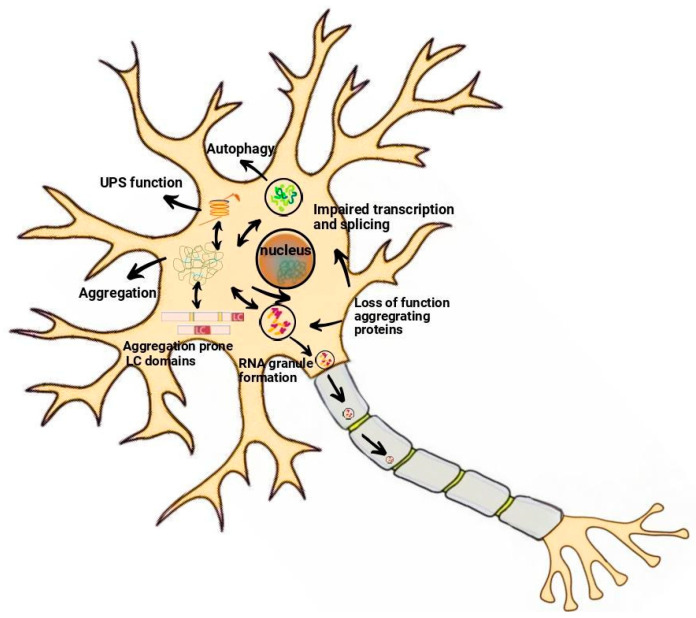
Complex neuronal mechanisms driving ALS progression and development.

Three theories explain ALS pathogenesis. The first, the dying forward hypothesis, posits that ALS starts in the cortex, affecting corticospinal motor neurons (MNs) linked to spinal cord MNs via monosynaptic connections. Glutamate excitotoxicity is believed to cause MN degeneration along the axon [16]. The second theory, the dying back hypothesis, suggests ALS begins with lower MN dysfunction, possibly originating from muscle or the neuromuscular junction. The retrograde transport of harmful substances leads to toxicity, potentially linked to axoplasmic transport dysfunction [17,18]. The third theory, the independent degeneration hypothesis, proposes autonomous degenerative changes in corticospinal and lower MNs, spreading along their neuroanatomical pathways [17]. ALS exhibits diverse characteristics, including varying corticospinal or lower MN involvement and distinct disease progression rates [19]. The precise pathophysiology of ALS is not fully understood, but multiple mechanisms have been associated with its development and progression (Figure 2).

Several molecular mechanisms underpin the pathophysiology of ALS. A primary mechanism involves mutations in the *SOD1* gene, leading to protein misfolding and aggregation. Mutant *SOD1* disrupts the redox balance, producing abnormal reactive oxygen species and reactive nitrogen species [20,21]. The accumulation of improperly folded proteins, including TDP-43 and SOD1, leadsto protein aggregation and neuronal toxicity [22]. SOD1 is transported to mitochondria by the translocase of the outer membrane complex despite lacking a mitochondrial localization signal. Within mitochondria, mutant *SOD1* builds up in the intermembrane space (IMS) and matrix, leading to toxic effects [23]. Misfolded SOD1 accumulates on the outer mitochondrial membrane (OMM) and contributes to apoptosis reliant on mitochondria. It is noteworthy that introducing external mutant *SOD1* aggregates has been shown to induce the misplacement of TDP-43 into the cytoplasm and intensify its aggregation [23].

Oxidative stress, mitochondrial dysfunction, and excitotoxicity contribute to neuronal damage and death in ALS. Elevated levels of metal ions can exacerbate these issues by inducing oxidative stress, mitochondrial dysfunction, protein misfolding, DNA damage, and endoplasmic reticulum stress, further advancing the progression of the disease [24,25,26]. Oxidative stress contributes to ALS development and motor neuron degeneration, leading to increased oxidative stress biomarkers in cerebrospinal fluid (CSF), plasma, and urine. Environmental factors can increase systemic oxidative stress, accelerating ALS progression [27,28].

Glial cell dysfunction, particularly involving astrocytes and microglia, also plays a crucial role in ALS pathophysiology through neuroinflammation and impaired support of neuronal function [29]. Genetic factors are implicated in both familial and sporadic forms of ALS, with mutations in genes such as *C9ORF72*, *SOD1*, *TARDBP*, and *FUS* contributing to disease susceptibility. 

While 90–95% of ALS cases are sporadic (sALS), 5–10% of familial ALS cases are linked to mutations in the *TARDBP* gene. The remaining 90–95% of familial cases are caused by mutations in other genes, such as *C9ORF72* (Hexanucleotide repeat expansion in *C9ORF72*), *SOD1*, *FUS*, and *NEK1* (NIMA-like kinase 1). Interestingly, up to 97% of sporadic ALS patients also have TDP-43 protein deposits in their neuronal inclusions, indicating a crucial role for TDP-43 in ALS pathology [30].

Dysfunction in RNA metabolism, protein homeostasis, and axonal transport further exacerbates motor neuron vulnerability in ALS. Defects in axonal transport and mitochondrial dysfunction play critical roles in motor neuron degeneration, further aggravating the disease progression [31]. RNA-binding proteins (RBPs) feature highly conserved RNA recognition motifs (RRMs) and play vital roles in various RNA metabolic processes, including mRNA processing, RNA export, and RNA stability. Certain RBPs, like TDP-43, are linked to neurodegenerative diseases, suggesting that disruptions in RNA metabolism may be a causative factor [32,33,34]. Two RRM domains of TDP-43 (RRM1 and RRM2), separated by 15 amino acids, bind RNA/DNA with a preference for UG/TG-rich sequences [35,36]. ALS-linked mutations P112H and D169G disrupt RNA binding without affecting recognition [37,38,39]. The RRM2 domain is suggested to contribute to TDP-43 protein dimerization.

### 2.1. Protein Aggregation

Protein aggregation is a defining feature of ALS, with essential proteins like SOD1, TDP-43, and FUS implicated. Misfolded proteins, such as TDP-43, SOD1, FUS, and others, accumulate in motor neurons, forming insoluble aggregates that disrupt cellular function, causing toxicity and cell death [40,41]. Mutant SOD1 proteins form toxic inclusions in motor neurons. TDP-43 and FUS aggregates disrupt RNA processing, causing neurodegeneration [42,43,44].

The exact mechanisms of protein aggregation in ALS are complex and intricate, involving the impairment of protein quality control mechanisms, such as proteasomal and autophagic degradation pathways. Alterations in RNA metabolism and stress granule dynamics contribute to the accumulation of misfolded proteins. Protein aggregation not only directly damages neurons but also triggers neuroinflammation and glial activation, further exacerbating neurodegeneration. These protein inclusions interfere with cellular functions, leading to motor neuron death and ALS progression [36]. (Table 1) represents the various genes involved in ALS pathogenesis.

### 2.2. Neuroinflammation

Neuroinflammation has a pivotal role in the pathophysiology of ALS, contributing to the progressive degeneration of motor neurons. Neuroinflammation in SALS and FALS involves reactive astrocytes, microglia, peripheral immune cells, and elevated levels of inflammatory mediators in CNS motor regions [56]. Recent studies indicate that activated microglia and astrocytes play significant roles in this process. Microglia, the primary immune cells in the central nervous system, become activated and release pro-inflammatory cytokines such as IL-1β, IL-6, and TNF-α,contributing to motor neuron damage [57]. Astrocytes also exhibit an abnormal inflammatory response, releasing toxic substances like ROS and further exacerbating neuronal injury [58].

In ALS, activated microglia and astrocytes secrete pro-inflammatory cytokines, chemokines, and ROS, creating a toxic microenvironment that exacerbates neuronal damage. This chronic neuroinflammatory response perpetuates a cycle of neuronal injury and glial activation, amplifying neurodegeneration. Dysregulated immune responses, including aberrant activation of the innate and adaptive immune systems, further contribute to neuroinflammation in ALS. Defects in the blood-brain barrier might allow peripheral immune cells to penetrate the CNS, thereby amplifying inflammatory responses. Targeting neuroinflammation can be a promising therapeutic approach for ALS, while aiming to modulate immune responses, mitigate glial activation, and promote neuroprotection [59]. Microglia are classified into M1 and M2 types. M1 microglia, induced by TLRs and gamma interferon signaling, produce pro-inflammatory cytokines (IL-1, IL-6, IL-1β, TNF-α, NF-kappaB) and chemokines, as well as NADPH oxidases and matrix metalloproteinase-12. In contrast, M2 microglia, associated with neuroprotection, express Arg-1, secrete growth factors, and release anti-inflammatory cytokines (IL-10 and TGF-β). This classification underscores the complex roles of microglia in regulating neuroinflammation and neuroprotection within the CNS [60,61,62,63].

The entry of peripheral immune cells, such as T-cells, into the CNS further amplifies neuroinflammation. This chronic inflammatory state disrupts the neuroprotective environment, leading to sustained neuronal stress and degeneration [64]. Therapeutic strategies targeting neuroinflammatory pathways, such as the modulation of microglial activation or the inhibition of specific cytokines, are being explored to potentially slow ALS progression and improve patient outcomes [65].

### 2.3. Oxidative Stress

Oxidative stress (OS) is a prominent feature in the pathophysiology of ALS, contributing to the progressive degeneration of motor neurons. Neuroinflammation and OS are intertwined in neurodegenerative diseases. OS arises from increased ROS and reduced antioxidant defenses. Glial and immune cells produce ROS and reactive nitrogen species (RNS), which potentially worsens ALS progression [66].

In ALS, an imbalance between ROS production and antioxidant defense mechanisms leads to cellular damage. Studies show that mutations in the *SOD1* gene lead to increased OS by allowing the accumulation of superoxide radicals [67].

Motor neurons are particularly susceptible to oxidative damage due to their high metabolic rate and limited antioxidant defense. Increased levels of ROS cause oxidative damage to proteins, lipids, and DNA, contributing to neuronal degeneration and cell death [68]. Also, mitochondrial dysfunction in ALS exacerbates oxidative stress by impairing the ETC, leading to excessive ROS production [39]. In addition, glial activation and neuroinflammation contribute to oxidative stress through the release of inflammatory mediators and ROS-producing enzymes [37]. Therapeutic approaches aimed at reducing oxidative stress, such as antioxidants and agents targeting mitochondrial function, are under investigation to potentially slow ALS progression [69].

### 2.4. Excitotoxicity

Excitotoxicity, characterized by excessive activation of glutamate receptors leading to neuronal damage, is a significant contributor to the pathophysiology of ALS. During glutamatergic transmission, presynaptic glutamate activates ionotropic receptors on the postsynaptic neuron. When glutamate receptors are activated, Na^+^ and Ca^2+^ ions enter the cell, causing depolarization and action potential. Over-stimulation leads to excitotoxicity, a form of neuronal degeneration [70]. Experimental evidence suggests excitotoxicity might contribute to neuronal damage associated with stroke, neurotrauma, epilepsy, and neurodegenerative disorders like ALS [71]. This process involves the excessive activation of glutamate receptors, particularly the N-methyl-D-aspartate (NMDA) receptors, leading to neuronal damage and death. In ALS, impaired glutamate uptake by astrocytes, due to the downregulation of the glutamate transporter EAAT2, results in elevated extracellular glutamate levels [72]. In ALS, dysregulation of glutamate homeostasis and impaired glutamate uptake by astrocytes result in excitotoxicity, primarily affecting motor neurons. Excessive glutamate signaling leads to sustained calcium influx, mitochondrial dysfunction, and the activation of cell death pathways, ultimately culminating in motor neuron degeneration. The calcium overload triggers the activation of proteases, lipases, and nitric oxide synthase, leading to oxidative stress and mitochondrial dysfunction. Glutamate excitotoxicity is further exacerbated by oxidative stress, protein aggregation, and neuroinflammation, creating a vicious cycle of neuronal damage in ALS [73]. These molecular disruptions contribute to the degeneration and apoptosis of motor neurons. Therapeutic strategies targeting excitotoxicity, such as the use of glutamate antagonists and drugs enhancing glutamate uptake, are being explored to mitigate motor neuron loss in ALS [74]. The drug riluzole, which modulates glutamate release, remains one of the few approved treatments shown to extend survival in ALS patients [10].

## 3. Biomarkers for Amyotrophic Lateral Sclerosis

The ALS biomarkers play a crucial role in the prediction and prognosis of disease development, and the selection of suitable treatment strategies can be madeby identifying the biomarkers of ALS [75]. Biomarkers of ALS are further subcategorized based on their primary function in the disease process. 

Diagnostic biomarkers are used to identify whether an individual is affected by a disease and indicates the possibility of a disease to develop in those individuals without any symptoms. Moreover, in patients with the disease diagnosed, the risk of future clinical events can be assessed using prognostic biomarkers, whereas, predictive biomarkers can help identify the specific treatments that provide the most benefit to patients. To measure the biological response to the treatment the patient is currently undergoing, response biomarkers are evaluated [76].

The biological markers can be analyzed in various biofluid samples, including the CSF, blood, urine, and saliva [76]. CSF samples require invasive collection; however, this provides direct access to the CNS. A blood sample involves easier collection, but its high protein levels complicates the diagnosis [77]. Urine samples, though easily accessible, provide limited and inconsistent information, while saliva is easy to collect and has scope for future research [76]. 

Neurofilaments, the neuronal cytoskeletal proteins that include light (NfL), medium (NfM), and heavy (NfH) chains, are the key biomarkers of ALS in the assessment of its progression, severity, and response to therapy [78]. These biomarkers can be detected in blood using the most advanced fourth-generation technologies [79]. The NfL levels in blood or CSF indicate ALS progression, severity, and survival. The NfL can help diagnose ALS early [80,81]. In the clinical trial conducted by ATLAS, elevated NfL levels were used to identify active disease in *SOD1* variant carriers [82]. Thus, it is a validated prognostic risk biomarker for ALS with potential applications in presymptomatic diagnosis and clinical trials.

ALS involves immune system dysregulation, including innate and adaptive immune systems. Initially, the immune system will be active, further as the disease progresses harmful pro-inflammatory phase sets in [83]. Studies report that inflammatory markers like C-reactive protein (CRP) and Interleukin-6 (IL-6) have shown inconsistent results in predicting disease progression [84,85]. However, markers such as neuroinflammatory proteins like NfL and chitinases (CHIT1 and YKL-40), individually as well as when combined, have shown better prognostic results [86]. Additionally, immunophenotyping studies and the analysis of particular immune cells, such as regulatory T cells, along with the detection of autoantibodies in ALS patients, provide insights into the progression and survival of the disease [87].

ALS is characterized by metabolic abnormalities, including disrupted lipid and glucose metabolism, hypermetabolism, and dysfunctional mitochondria [88]. Cholesterol levels, especially HDL-C and LDL-C have shown inconsistent results in predicting ALS prognosis [89]. However lipidomics, an emerging technology, identifies more reliable lipid-based biomarkers such as ganglioside GA2 and ganglioside GM3 [90]. The elevated serum glucose levels and ferritin levels due to impaired glucose and iron metabolism respectively are linked to increased disease risk and mortality [91,92]. On other hand, a lower serum albumin level has been correlated with inflammation and worse outcomes [93].

Muscle damage markers like plasma creatinine and creatine kinase are promising biomarkers for ALS, reflecting disease severity and muscle denervation. Plasma creatinine serves as a prognostic tool as it has shown strong correlations with muscle strength, ALSFRS-R scores, and survival [94]. The decline of creatine kinase levels at diagnosis over time correlates with disease aggressiveness and survival [95]. Further, rising levels of cardiac troponin T (cTnT) arelinked to disease progression [96]. In addition to tracking key biomarkers like neurofilaments and inflammation, examining ALS-related genes, RNA-binding proteins (RBPs), and non-coding RNAs offers a more nuanced perspective on the genetic and molecular drivers of ALS.

RBPs indicate the importance of RNA metabolism and protein aggregation in ALS pathology and its progression [97]. The deficiency of FUS due to aggregation and abnormal phase transition causes neuronal cell death, contributing to ALS [98]. The mutation of FET family proteins such as TAF15 and EWSR1 also leads to neurodegeneration and ALS progression. ATXN2, involved in RNA metabolism, has CAG repeat expansions and is associated with ALS [99,100]. ATXN2 and hnRNPs, such as hnRNPA1 and hnRNPA2/B1, interact with TDP-43, causing mutations in their prion-like domains triggering proteinopathies and ALS [101,102]. The mutation and mislocalization of thenuclear matrix protein MATR3 arelinked to ALS [103].

Non-coding RNAs, including circular RNAs (circRNAs), microRNAs (miRNAs), and long non-coding RNAs (lncRNAs), serve as potential ALS biomarkers. The altered levels of MiRNAs like miR-27a and miR-124 are linked to ALS progression [104]. LncRNAs interact with ALS-related proteins such as TDP-43 and FUS, possibly contributing to the disease [105]. The increased levels of CircRNAs have been identified as potential blood-based biomarkers for ALS [106].

## 4. Emerging Therapeutic Targets for ALS

Treatment strategies for ALS are evolving, focusing on various approaches to slow disease progression and improve patient outcomes. Key therapeutic strategies include inhibiting protein aggregation using small molecules, modulating neuroinflammatory pathways to reduce neuronal damage, and employing antioxidant therapies for mitochondrial protection. Currently, precision medicine and personalized approaches such as gene therapy and stem cell therapy offer the potential to revolutionize ALS treatment. However, breakthroughs in genetics, biomarker discovery, and molecular profiling are enabling personalized treatments tailored to an individual’s molecular and genetic makeup. This allows for targeted therapies that address specific genetic abnormalities, enhance disease monitoring, guide treatment selection, and improve patient stratification [107].

### 4.1. Inhibiting Protein Aggregation by Small Molecules

Protein aggregation, a key pathological feature of ALS, has been shown to be inhibited by several small molecules to mitigate neurodegeneration. These small molecules signify a diverse range of strategies to combat protein aggregation in ALS, such as stabilization of native protein structure and increased autophagic clearance, each offering a unique mechanism of action with potential therapeutic benefits. Several small molecules have been identified and are being studied for their potential to prevent or interfere with the aggregation of proteins like TDP-43, SOD1, and others linked to ALS [108].

A novel therapeutic approach involves the application of small molecules, like molecular tweezers, which selectively bind to lysine and arginine residues to prevent protein aggregation. These molecules preserve the bioactivity of proteins while preventing the abnormal interactions that lead to their aggregation. For example, CLR01 is one of the most studied molecular tweezers and has demonstrated a reduction in SOD1 aggregation within the spinal cords of mouse models. However, it is important to emphasize that CLR01 did not lead to an improvement in the motor function of the mice [108]. Samanta et al. (2020) have also demonstrated that CLR01 intervenes at the initial stage of the aggregation process, preventing the unfolding of the SOD1 monomer by using a truncated version of the wild-type SOD1 protein [109]. Further research is essential to understand the potential of CLR01 as a therapeutic candidate for ALS.

Epigallocatechingallate (EGCG), a small molecule derived from green tea, also exhibits a similar mechanism by stabilizing proteins and preventing aggregation. In vivo studies using the SOD1-G93A mouse model have demonstrated that oral administration of EGCG significantly delays the onset of symptoms and extends survival in ALS mice [110]. Pyrazolone derivatives have also been shown to inhibit protein aggregation, resulting in improved motor function and extended survival in ALS mouse models [111]. Lipoamide, a molecule targeting protein aggregation, modulates stress granule proteins like FUS and TDP-43, suggesting a potential for slowing ALS progression [112]. However, extensive testing in various ALS models is required to determine their therapeutic efficacy for ALS treatment.

### 4.2. Modulation of Neuroinflammatory Pathways

The suppression of microglial activation, particularly through modulation of neuroinflammatory pathways, is a promising area of research for various neurological conditions. Some inhibitors target signaling pathways or specific receptors involved in microglial activation through various mechanisms, such as the nuclear factor kappa B (NF-κB) signaling pathway, toll-like receptor (TLR) pathways, or the purinergic receptors, while others may alter the release of certain pro-inflammatory cytokines. In ALS mouse models, inhibiting the microglial activation is a crucial aspect of therapeutic intervention. For example, in hSOD1G93A transgenic mice, diphenyl diselenide (DPDS) suppresses microglial activation by blocking the NLRP3 inflammasome and the IκB/NF-κB pathways, thereby reducing the neuroinflammation [113]. In another study, reduced reactive microglia and prolonged survival wereobserved in SOD1G93A mice when orally administered with Nitroalkene Benzoic Acid Derivative (BANA) on a daily basis [114]. Receptor-interacting serine/threonine protein kinase 1 (RIPK1) is a protein that is activated by TNF-α via TNF receptor 1, and its activation can enhance microglial activity and induce cell death. SAR443820, an RIPK1 inhibitor, has demonstrated the ability to reduce microglial inflammation by effectively binding to the RIPK1 [115]. Another promising strategy that is being tested in ALS subjects is the use of the small molecule BLZ945 to modulate microglia. As a CSF1R kinase inhibitor, BLZ945 has been demonstrated to deplete microglia and enhance remyelination in the cortex and striatum in mice [116,117]. Csf1R levels are known to be significantly elevated in microglia in ALS mice compared to healthy mice. Inhibiting Csf1R with the small molecule GW2580 substantially reduces microglial proliferation and the number of CD68+ activated microglia in the spinal cord. This inhibition slows disease progression, highlighting the critical role of CSF1R signaling in ALS pathobiology [118]. Drugs with various mechanisms and molecular targets are being studied in clinical trials. Therapeutic agents like masitinib, ibudilast, and NP001 have been reported to primarily affect microglia by blocking the production of key molecules involved in the inflammatory response [119].

### 4.3. Antioxidant Therapies and Mitochondrial Protection

Antioxidants are molecules that combat oxidative stress by protecting cells from damage caused by free radicals. They play a crucial role in treating neurodegenerative diseases like ALS, where oxidative stress contributes to neuronal death [120]. Several molecules with antioxidant potential have been identified, including vitamin E, flavonoids, carotenoids, resveratrol, curcumin, coenzyme Q10, melatonin, and edaravone. Vitamin E, a lipophilic antioxidant, has been associated with a delay in the clinical onset of the disease in the *SOD1* mutant mouse model [121]. Additionally, by increasing plasma glutathione levels, Vitamin E enhances the systemic antioxidant defense mechanisms in patients with ALS [122]. Another study observed that individuals with increased levels of vitamin E might have a reduced risk of ALS-related mortality [123]. Plant pigments such as astaxanthin and lycopene have demonstrated benefits in ALS due to their antioxidant properties [124]. Consequently, consuming carotenoids might help prevent ALS and/or delay its onset [125] and could potentially act as therapeutic agents for addressing neuroinflammation and apoptosis in ALS patients [126]. Plant flavonoids like salvianolic acid A, 7,8-dihydroxyflavone (7,8-DHF), fisetin, and quercetin show therapeutic potential against ALS by improving motor deficits and reducing ROS levels. Salvianolic acid A inhibits SOD1 aggregation and enhances stability, making it promising for treating the D124V mutant [127,128,129]. Curcumin from turmeric has also shown neuroprotective effects in ALS by reducing oxidative stress, inflammation, and protein aggregation. It activates *Nrf2* genes, reduces ROS, and mitigates TDP-43 excitability and mitochondrial dysfunction [130,131,132,133]. Antioxidants such as resveratrol upregulate *sirtuin 1* (*SIRT1*), delay ALS onset, and enhance motor neuron survival [134,135], while Coenzyme Q10 extends survival in ALS mice and increases brain mitochondrial levels, despite inconsistent correlations with ALS risk [136,137,138]. High oral doses of melatonin slowed disease progression and improved survival rates in the SOD1G93A transgenic mouse model of ALS [111,139].While edaravone functions as a free radical scavenger, it may potentially slow disease progression with its safety established in ALS patients [140].

### 4.4. Gene Therapy Approaches

In recent years, gene therapy has made remarkable progress by altering gene expression or modifying cell properties for therapeutic purposes. This progress has sparked interest in treating motor neuron diseases (MNDs), with several potential molecular targets identified. Some of the key methods include RNA-modifying therapies using antisense oligonucleotides (ASOs) and small interfering RNAs (siRNAs), as well as viral vectors. RNA interference (RNAi) is promising for treating SOD1 ALS by targeting RNA/protein-related toxicity. Unlike ASOs, RNAi uses double-stranded RNAs processed enzymatically to suppress gene expression through the RNA-induced silencing complex (RISC). Methods like siRNAs, shRNAs, and artificial miRNAs are delivered via AAV vectors for efficacy. In SOD1G93A mouse models, AAV-mediated siRNA delivery extended survival by 39%, with efficacy declining with age [141]. Initial studies demonstrated that delivering siRNA via AAV-2-mediated retrograde transport from muscles to spinal motor neurons reduced SOD1 levels in ALS mouse models [142]. Rizvanov et al. (2009) reduced human SOD1 mRNA levels in the lumbar spinal cord by 48% by delivering siRNA directly to the sciatic nerve in mice [143].

In 2017, AAV10 vectors were employed for hSOD1 pre-mRNA exon skipping, resulting in reduced SOD1 levels through alternative splicing. This treatment, delivered intravenously and intraventricularly, prevented weight loss, maintained neuromuscular function, and increased survival in mice, indicating its potential effectiveness [144]. However, challenges such as in vivo instability, siRNA specificity, and potential toxicity remain for RNAi-based gene therapy in ALS. On the contrary, ASOs are short single-stranded nucleic acids that specifically bind mRNA to alter processing or induce degradation through RNase H.

ASOs degrade mRNA via RNase H1 in both cytoplasm and nucleus, reducing protein synthesis, beneficially impacting neurodegenerative diseases. In ALS, intrathecal ASO injections targeting *SOD1* into rats and rhesus monkeys demonstrated CNS penetration and slowed disease progression in animal models [48]. In humans, phase I trials confirmed safety, but Phase III trials with tofersen (BIIB067) did not meet primary ALS endpoints, necessitating further study [145]. *C9orf72*-ALS, targets RNA expansions, showing efficacy in mice and is progressing to Phase I trials (NCT03626012) [BIIB078193] [146].

ASOs targeting *SOD1* and *C9orf72* in ALS have shown efficacy in reducing mRNA and protein levels and are progressing to clinical trials [147]. These therapies target specific ALS mutations and expansions, aiming to mitigate disease progression effectively. CRISPR-Cas9 genome editing shows promise for treating genetic and non-genetic disorders, particularly ALS. Studies using AAV vectors to deliver CRISPR-Cas9 have successfully reduced mutant SOD1 and C9orf72 protein levels, improving motor function in mouse models. However, challenges like off-target effects and delivery methods remain. Enhancing small guide RNA (sgRNA) specificity and exploring non-viral delivery systems are ongoing efforts. A study by Gaj et al. (2017) demonstrated significant benefits in early-stage ALS mice using the AAV-SaCas9-sgRNA system, and their results showed improved life expectancy by 54.6% [148]. Similarly, Duan et al. (2019) demonstrated that CRISPR-Cas9 prevented disease progression in *SOD1* mice using AAV-SaCas9-sgRNA to delete the *SOD1* gene in G93A-SOD1 mice [149]. Researchers conducted studies on *C9orf72*, using CRISPR-Cas9 to eliminate the large repeat expansion in *C9orf72* from patient-derived iPSCs. This approach prevented RNA foci and hypermethylation without affecting *C9orf72* expression [150]. Similarly, Lopez-Gonzalez et al. (2019) deleted G4CA repeats in *C9orf72* iPSCs, reducing overactive DNA repair and pro-apoptotic protein expression [151]. Future research aims to expand targets and address ethical and safety concerns for clinical use.

### 4.5. Stem Cell-Based Therapies for Neuroprotection and Regeneration

Stem cell therapy, a type of regenerative medicine, utilizes stem cells to repair or replace damaged tissues and cells in the body, aiming to slow or reverse the progression of ALS. Stem cells possess the unique ability to differentiate into different cell types, and they can also self-renew to generate additional stem cells. The two main types of stem cells utilized in ALS treatment are neural stem cells (NSCs) and mesenchymal stem cells (MSCs) [152]. NSCs are pluripotent stem cells that can differentiate into various cell types in the nervous system, including neurons, astrocytes, and oligodendrocytes, while MSCs are multipotent cells capable of differentiating into various mesenchymal tissues, including bone, cartilage, muscle, and fat. NSCs are typically transplanted surgically into the spinal cord or brain ventricles, while MSCs could be administered through intravenous or intraspinal injections. To assess the safety and effectiveness of NSCs and MSCs in ALS patients, many clinical trials are either in progress or have been completed. Pre-clinical studies using MSCs on *SOD1* mutant SOD1-G93A mouse/rat have demonstrated a delay in motor neuron degeneration, improved motor function, and an extended life span [152,153]. In 2003, the first clinical trial was conducted by Mazzini et al. using MSCs to treat ALS. In this study, autologous bone marrow MSCs were administered to seven patients through intrathecal injections [154]. The cells were welltolerated, with no serious side effects or abnormal spinal cord changes observed. However, no results on effectiveness were reported due to the absence of a control group, though the study provided hope for future research. Subsequent studies by Mazzini reported similar safety results, including a 9-year follow-up showing no serious side effects and some patients experiencing slower disease progression and increased life expectancy [155]. Later, Mazzini et al. (2012) conducted transplantation of stem cells in 18 ALS patients by microinjecting human neural stem cells (hNSCs) into the lumbar spine or cervical medullary gray matter. During the 60-month observation period, the disease progression was monitored without detecting any serious adverse effects, while some patients exhibited temporary clinical improvement [156].

In a study carried out by Petrou et al. (2021), no adverse effects were detected during the trial when 20 ALS patients with ALSFRS-R scores >20 received 1–4 intrathecal MSC injections at 3–6 month intervals [157]. Only two clinical trials, which adhered to low-bias randomized controlled trial (RCT) guidelines (NCT01363401 and NCT03280056), have been conducted. The phase II RCT lenzumestrocel (NCT01363401) showed temporary improvements in ALSFRS-R score with good safety profiles. Here, the patients received only 2 intrathecal injections of autologous BM-MSCs with riluzole alone as controls. In the NurOwn phase III RCT (NCT03280056), patients were administered three intrathecal injections of autologous preconditioned BM-MSCs or a placebo. The trial did not meet its primary outcome. However, subgroup analysis in patients with early and moderate stages of ALS indicated potential clinical benefits based on ALSFRS-R scores. The study also maintained good safety standards [158]. Despite showing safety and efficacy in ALS clinical trials, stem cell therapy faces challenges. Effectiveness varies due to multiple factors, and uniform standards are lacking. Risks include financial burden, cell supply issues, and heterogeneity, complicating stem cell transplantation. The advantages and disadvantages of various therapeutic approaches are listed in Table 2.

## 5. Conclusions and Prospects

ALS remains a damaging neurodegenerative disorder with significant clinical and therapeutic challenges. This review has provided a comprehensive overview of ALS, highlighting its epidemiology, pathology, and clinical symptoms. The necessity of developing effective therapies is underscored by the progressive and fatal nature of the disease, necessitating a deep understanding of its complex pathophysiology. The pathophysiology of ALS involves a confluence of molecular mechanisms, including protein aggregation, neuroinflammation, oxidative stress, and excitotoxicity. These interconnected pathways contribute to motor neuron degeneration and the progressive loss of muscle function. Advancements in the understanding of these mechanisms have facilitated the identification of potential biomarkers, offering hope for earlier diagnosis and a more precise monitoring of disease progression.

Emerging therapeutic targets present new prospects for ALS treatment. Inhibiting protein aggregation with small molecules, modulating neuroinflammatory pathways through inhibitors of microglial activation, and utilizing antioxidant therapies to protect mitochondria represent promising strategies. Gene therapy approaches, such as RNA interference and gene editing, along with stem cell-based therapies aimed at neuroprotection and regeneration, also show significant potential. Precision medicine and personalized approaches are poised to revolutionize ALS treatment, customizing treatments to specific genetic and molecular profiles. As research continues to unravel the complexities of ALS, these innovative strategies are expected to enhance therapeutic efficacy and improve patient outcomes. Future studies should focus on translating these findings into clinical practice, optimizing existing therapies, and developing novel interventions that target the root causes of ALS.

## Figures and Tables

**Table 1:** Key genes and proteins associated with ALS and their functional roles.

Gene/Protein	Role of Gene/Protein	Association with ALS	Reference
Annexin A11 (*ANXA11*)	Phosphoinositide-binding protein involved in RNA transport and lysosome interaction	Mutations impair RNA transport, triggering neuronal apoptosis. The abnormal protein aggregation linked to ALS	[45]
*C21orf2*	Interacts with NEK1, involved in microtubule assembly, DNA repair, and mitochondrial function	Interrelated with C9orf72 and NEK1 mechanisms, thereby the mutation is associated with ALS	[46]
*C9orf72*	Involved in vesicle transport, lysosomal homeostasis, mTORC1 signaling, and autophagy	The repeated expansion of GGGGCC causes neuroinflammation leading to familial ALS (fALS)	[47]
*CHCHD10*	Mitochondrial protein linked to clinical lineage of ALS-FTD (frontotemporal dementia)	Mitochondrial dysfunction may play a role in ALS and FTD pathogenesis	[48]
Cyclin F (*CCNF*)	Part of E3 ubiquitin-protein ligase complex	Mutations increase TDP-43 aggregates, leading to ALS	[49]
Glycosyltransferase 8 domain 1 (*GLT8D1*)	Encodes glycosyltransferase, potentially toxic when mutated	Mutation of glycosyltransferase is associated with FALS development	[50]
Kinesin family member 5A (*KIF5A*)	Member of kinesin family, involved in cargo transport	Mutation in the C-terminal cargo-binding tail domain of KIF5A	[51]
NIMA-related kinase 1 (*NEK1*)	Kinase involved in cell cycle progression and mitosis	Variants of this kinase areassociated with fALS risk	[52]
*SPG11* (spatacsin)	Key role in axon maintenance, synaptic vesicle transport, and autophagy	Mutations cause juvenile ALS with slower progression than adult ALS	[53]
Superoxide Dismutase 1 (*SOD1*)	Powerful antioxidant enzyme that protects against superoxide free radicals	170 mutations linked to ALS; misfolded SOD1 spreads in a prion-like manner; causes motor neuron death and enhanced apoptosis. Early ER–Golgi transport dysfunction in mice	[54]
TANK-binding kinase 1 (*TBK1*)	Member of IκB kinase family, involved in innate immune signaling	Linked to TDP-43 proteinopathies. Insufficient TBK1 function causes ALS and FTD	[55]

**Table 2:** Main advantages and limitations of the different therapeutic approaches in Amyotrophic Lateral Sclerosis.

	Therapy	Advantages	Disadvantages/Limitations	References
Small Molecules	Molecular tweezers—CLR01	Reduces the aggregation of SOD1 in the spinal cord of mouse models.	Did not improve the motor function of the mice.	[108]
	Epigallocatechingallate (EGCG)	Significant delay in the onset of symptoms and prolonged survival in ALS mice.	Extensive testing in ALS models is still required for therapeutic efficacy.	[110]
	Pyrazolone derivatives	Improved motor function and extended survival in ALS mouse models.		[111]
	Lipoamide	Modulates stress granule proteins such as FUS and TDP-43.		[112]
Microglial Activation Inhibitors	Diphenyl diselenide (DPDS)	Suppresses microglial activation by inhibiting the NLRP3 inflammasome and the IκB/NF-κB pathways.	Understanding the regulation and release of microglial-associated inhibitors is crucial for assessing therapeutic potential.	[113]
	Nitroalkene Benzoic Acid Derivative (BANA)	Reduced reactive microglia and prolonged survival in SOD1G93A mice.		[114]
	RIPK1—SAR443820	Reduces microglial inflammation.		[115]
	CSF1R kinase inhibitor, BLZ945	Depletes microglia and enhances remyelination in the cortex and striatum in mice.		[116,117]
	GW2580	Reduces microglial proliferation and slows disease progression.		[118]
	Drugs—masitinib, ibudilast, and NP001	Target microglia by inhibiting the production of molecules that play a crucial role in the inflammatory response.		[119]
Antioxidant Therapies and Mitochondrial Protection	Vitamin E	Delays the onset of disease in the SOD1 mutant mouse model. Enhances the systemic antioxidant defense mechanisms in patients with ALS. Reduced risk of mortality in ALS patients.	Need for more studies due to the limited availability of results, which are often contradictory, inconclusive, or statistically insignificant.	[121,122,123]
	Plant pigments—astaxanthin and lycopene	Antioxidant properties.		[124]
	Carotenoids	Prevent ALS and/or delay its onset. Therapeutic molecule for treating neuroinflammation and apoptosis in ALS patients		[125,126]
	Flavonoids—Fisetin, and quercetin	Improves motor deficits and reduces ROS levels. Inhibits SOD1 aggregation and enhances stability		[127,128,129]
	Salvianolic acid A, 7,8-dihydroxyflavone	Inhibits SOD1 aggregation and enhances stability		[129]
	Curcumin	Reduces oxidative stress, inflammation, and protein aggregation		[130,131,132,133]
	Resveratrol	Upregulates sirtuin 1 (SIRT1), delays ALS onset, and enhances motor neuron survival.		[134,135]
	Coenzyme Q10	Extends survival in ALS mice and increases brain mitochondrial levels.		[136,137,138]
	Melatonin	Delayed disease progression and improved survival rates in the SOD1G93A transgenic mouse model.		[111,139]
Gene Therapy	AAV-mediated siRNA delivery	Reduced SOD1 levels in ALS.	Challenges such as in vivo instability, siRNA specificity, and potential toxicity remain for RNAi-based gene therapy.	[141,142,143,144]
	Antisense oligonucleotides (ASOs)	Slowed disease progression in animal models.		[48]
	CRISPR-Cas9	Prevented disease progression in *SOD1* mice using AAV-SaCas9-sgRNA. Improved life expectancy by 54.6%.	Ethical and safety concerns.	[148,149]
Stem Cell-based Therapies	MSC-SOD1G93A mice model	Delay in motor neuron degeneration, improved motor function, and extended lifespan.	Limited migration into CNS.	[152]
	Autologous bone marrow MSCs	Linear decline in FVC and ALSFRS was noted.	Absence of control group and small sample size.	[155]
	Autologous BM-MSCs	Showed temporary improvements in ALSFRS-R score with good safety profiles.	Limited sample size and the heterogeneity of individual disease progression.	[157]
	NurOwn^®^ autologous MSCs	Safe, increase of neurotrophic factors.	The trial did not meet its primary outcome.	[158]
